# Pattern of F-18 FDG Uptake in Colon Cancer after Bacterial Cancer Therapy Using Engineered *Salmonella Typhimurium*: A Preliminary *In Vivo* Study

**DOI:** 10.1155/2022/9222331

**Published:** 2022-04-19

**Authors:** Ari Chong, Dinh-Huy Nguyen, Hyeon Sik Kim, June-Key Chung, Jung-Joon Min

**Affiliations:** ^1^Department of Nuclear Medicine, Chosun University Medical School, Gwangju, Republic of Korea; ^2^Department of Nuclear Medicine, Chonnam National University Medical School, Jeonnam 519-763, Republic of Korea; ^3^Department of Nuclear Medicine, Chonnam National University Hwasun Hospital, Jeonnam 519-763, Republic of Korea; ^4^Medical & Bio Photonics Research Center, Korea Photonics Technology Institute, Gwangju, Republic of Korea; ^5^Department of Nuclear Medicine, Seoul National University College of Medicine, Republic of Korea

## Abstract

**Purpose:**

Bacterial cancer therapy (BCT) research using engineered *Salmonella typhimurium* has increased in recent years. 2-Deoxy-2[^18^F] fluoro-D-glucose positron emission tomography (FDG PET) is widely used in cancer patients to detect cancer, monitor treatment responses, and predict prognoses. The aim of this pilot study was to investigate FDG uptake patterns in a mouse tumor model after BCT. *Procedures*. BCT was performed via the intravenous injection of attenuated *S. typhimurium* (SL*Δ*ppGpp/lux) into female mice bearing a tumor (derived from CT26 murine colon cancer cells) in the right thigh. ^18^F-FDG PET images acquired before BCT and at different time points after BCT. *In vivo* bioluminescence imaging confirmed bacterial presence in the tumor. The tumor volume, standardized uptake value (SUV) of FDG (SUVmax and SUVmean), early SUV reduction%, and normalized tumor volume change were analyzed.

**Results:**

Early after BCT (1 or 2 days post-injection (dpi)), FDG tumor uptake decreased in 10 out of 11 mice and then increased at later stages. FDG uptake before BCT was correlated with normalized tumor volume change after BCT. Early FDG reduction% after BCT was correlated with normalized volume change after BCT.

**Conclusions:**

Early after BCT, FDG tumor uptake decreased and then increased at later stages. The higher the FDG tumor uptake before BCT, the better the BCT response. FDG uptake patterns were related to tumor volume change after BCT. Therefore, FDG uptake was a good candidate for evaluating BCT.

## 1. Introduction

Despite recent and considerable advances in cancer therapy, several major treatment limitations still persist, including drug resistance, limited effective treatment of metastatic tumors, and toxicity toward normal tissue [1]. As a result, alternative treatments have been suggested. Bacterial cancer therapy (BCT) is one such alternative; while its origins date back approximately 150 years, contemporary therapies involve the use of *Streptococcus pyogenes* to treat inoperable sarcoma [2]. Several other bacterial strains are also used for BCT [2–9], including *Escherichia coli* [5], *Clostridium* [10], *Salmonella* [3], *Listeria* [11], and *Bifidobacterium* [12], where the bacteria tend to accumulate in a broad range of tumors [7]. Several studies have reported tumor suppression after BCT [5, 6, 13]. Importantly, BCT studies are no longer limited to animal models; several clinical phase I–III trials have been conducted in cancer patients [9, 14–19].

A common bacteria strain for BCT is attenuated *Salmonella typhimurium* which is defective in *ppGpp* synthesis (*ΔppGpp S. typhimurium*) [20]. The bacteria displays a 100,000–1,000,000-fold increased median lethal dose [21]. Zheng et al. reported that *ΔppGpp S. typhimurium* was more than 10,000-fold higher in tumor tissue when compared with other organs [8]. Critically, bacteria injected during BCT were rapidly cleared from internal organs [5], while after tumor targeting, BCT bacteria were not released from tumors and did not re-enter the blood circulation [22].

Our previous *S. typhimurium in vivo* research showed that tumor growth after BCT exhibited two phases: tumor growth suppression for 1–10 days, but regrowth thereafter [23]. The trajectory of individual tumor growth after BCT often shows significant variations in treatment responses [24]. However, no modalities are currently available to predict the tumor suppression effects of BCT; therefore, *in vivo* imaging techniques are required to monitor and predict BCT effects.

The glucose analog, 2-deoxy-2[^18^F]fluoro-D-glucose (FDG) is widely used as a radiotracer for positron emission tomography (PET) and typically shows high FDG uptake in malignant tumors due to increased anaerobic glycolysis [25]. In patients with various cancers, FDG PET/computed tomography (PET/CT) strategies are used for tumor detection, monitoring treatment responses, and prognosis predictions. FDG also accumulates in activated immune cells [26], whereas BCT mechanisms trigger immune responses [7, 23, 27]. Therefore, based on these uptake modalities, FDG appears to be a good candidate for evaluating BCT. To our knowledge, no previous reports have analyzed FDG uptake changes in tumors after BCT.

We therefore investigated FDG uptake patterns in tumors in a murine tumor model using attenuated *S. typhimurium* during and after BCT.

## 2. Materials and Methods

### 2.1. Study Overview

**Figure 1:**
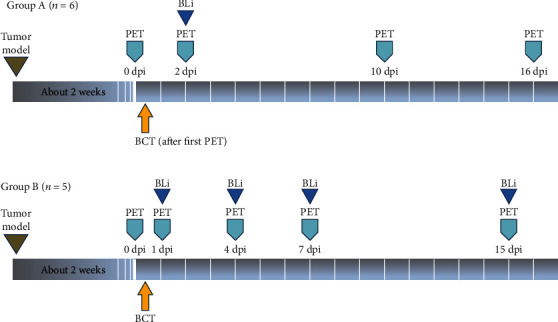
Study overview. This pilot study was performed using two separate trials: A and B. A tumor was generated in the right thigh of each mouse (BALB/c female mice, 6-weeks-old) using the murine CT26 colon adenocarcinoma cells. After tumor volume reached approximately 180 mm^3^, BCT was performed by intravenous injection of bacterial cells via the tail vein (bacterial dose = 4.5 × 10^7^ CFU/mouse in 100 *μ*L PBS). Two trials were designed to avoid animal preparation issues such as fasting and general anesthesia. In trial A (*n* = 6), FDG PET/CT images were acquired at 0 dpi, 2 dpi, 10 dpi, and 16 dpi (a). In trial B (*n* = 5), FDG PET/CT images were acquired at 0 dpi, 1 dpi, 4 dpi, 7 dpi, and 15 dpi (b). *In vivo* BLi confirming the presence of tumor-targeting bacteria was performed at 2 dpi, immediately after PET/CT in trial A, and immediately after each PET/CT in trial B. Abbreviations: FDG, 2-deoxy-2[^18^F]fluoro-D-glucose; dpi, days post-injection; SUVmax, maximum standardized uptake value; SUVmean, mean standardized uptake value; PBS, phosphate-buffered saline; CFU, colony-forming units; BLi, bioluminescence imaging.

This study was performed using two separate trials: experiment A (*n* = 6 mice) and B (*n* = 5 mice). We used this approach to avoid overburdening individual animals with different preparation procedures, such as fasting and general anesthesia (Figure 1). After generating colon cancer animal model (murine CT26 colon adenocarcinoma cell line and BALB/c female mice; Section 2.2), BCT was initiated using a single intravenous injection (Section 2.3). PET/CT scans were acquired before BCT and serially during follow-up.

In trial A, PET/CT scans were acquired at 0 days post-injection (dpi), 2 dpi, 10 dpi, and 16 dpi (a1–a6 mice).

In trial B, PET/CT scans were acquired at 0 dpi, 1 dpi, 4 dpi, 7 dpi, and 15 dpi (b1–b5 mice). The tumor size was measured at 0 dpi, 3 dpi, 6 dpi, 9 dpi, 12 dpi, 16 dpi, and 19 dpi in trial A and at 0 dpi, 3 dpi, 6 dpi, 9 dpi, 12 dpi, 15 dpi, and 17 dpi in trial B.

### 2.2. The Tumor Cell Line and the Mouse Model

In total, 11 female BALB/c mice (6-weeks-old) were used in this study. The murine CT26 colon adenocarcinoma cell line (American Type Culture Collection, CRL-2638) was xenografted into animals. Cells were cultured in Dulbecco's Modified Eagle Medium at 37 °C in 5% CO_2_ and used under specific pathogen-free conditions. A tumor was generated via the subcutaneous injection of 1 × 10^6^ CT26 cells in phosphate buffered saline (PBS) into the right thigh of each mouse. BCT was then initiated when the tumor volume reached approximately 180 mm^3^. Tumor length (L), width (W), and depth (D) dimensions were measured using a single caliper (mm^3^) by one researcher. Tumor volume (V) is calculated using the formula: *V* = (*L* × *W* × *W*)/2.

### 2.3. The Bacterial Strain and BCT

Attenuated *S. typhimurium* defective in *ppGpp* synthesis (RelA::cat, SpoT::kan) and expressing the bacterial luciferase (lux) operon (SHJ2037) (SL*Δ*ppGpp/lux) [28, 29] were used for BCT, which was performed using a one-time intravenous injection via the tail vein (bacterial dose = 4.5 × 10^7^ colony-forming units [CFU]/mouse in 100 *μ*L PBS [29]).

### 2.4. PET/CT Scanning

Six hours before the FDG injection, the mice were caged separately and all food and sawdust removed. Mice were masked and anesthetized using 2.5% isoflurane in air. Before the FDG injection, serum glucose levels were measured using a glucometer (Accu-Chek® Performa, Roche Diagnostics, Indianapolis, IN, USA) to ensure a fasting status. Then, ^18^F-FDG (7.4 MBq) was injected via the tail vein. After 1 h, PET/CT scanning was performed except head area. We used a microPET scanner (Inveon, Siemens Medical Solutions, Knoxville, TN, USA). Acquired images were reconstructed using a three-dimensional ordered-subset expectation maximization algorithm, with four iterations. The reconstructed pixel size was 0.78 mm in axial and transverse directions (128 × 128 pixels in each of 159 transverse slices). Data normalization, decay corrections, and dead time corrections were also performed. PET/CT images were analyzed using PMOD v.3.310 (PMOD Technologies, Ltd., Zurich, Switzerland). FDG uptake in tumors was measured as the standardized uptake value (SUV) [30, 31]. After manually drawing a region-of-interest (ROI) in the tumor at the highest uptake point, maximum standardized uptake (SUVmax) and mean standardized uptake values (SUVmean) were obtained using PMOD.

### 2.5. *In Vivo* Bioluminescence (BLi) Imaging

*In vivo* BLi confirmed successful injection and SL*Δ*ppGpp/lux accumulation in tumors. Images were acquired after PET/CT scanning on the same day. To capture images, an IVIS-100 imaging system equipped with a charged coupled device camera (Caliper Life Sciences, Waltham, MA, USA) was used. After anesthetization with 2.5% isoflurane in air, mice were placed in the light-tight chamber of the IVIS-100 system. Living image software v.2.25 (Caliper Life Sciences) was used for image acquisition and processing. In trial A, *in vivo* BLi was acquired at 2 dpi. In trial B, *in vivo* BLi was performed on the same day as PET scanning and *in vivo* BLi counts were also acquired.

### 2.6. Analysis of Variables

SUVmax, SUVmean, and tumor volume values at each time point were used for analyses. The following formula is used:

(1) $$\begin{array}{c} \text{SUV}=\frac{decay-corrected\;activity\left[kBq\right]/mL\;\text{of }tissue\;volume}{injected\;F-18\;FDG\;activity\;\left[kBq\right]/g\;\text{of }body\;mass}. \end{array}$$

A circular ROI was drawn over the FDG uptake area in the tumor, after which SUVmax and SUVmean values were obtained.

In addition, early SUVmax reduction%, early SUVmean reduction%, normalized tumor volume change, and growth ratio are calculated as follows:

(2) $$\begin{array}{c} \text{Early SUVmax reduction}\%=100\times \frac{SUVmax\left(0\;dpi\right)-SUVmax\;\left(1\;\text{or }2\;dpi\right)}{SUVmax\;\left(0\;dpi\right)}, \end{array}$$

(3) $$\begin{array}{c} \text{Early SUVmean reduction}\%=100\times \frac{SUVmean\left(0\;dpi\right)-SUVmean\;\left(1\;\text{or }2\;dpi\right)}{SUVmean\;\left(0\;dpi\right)}, \end{array}$$

(4) $$\begin{array}{c} \text{Normalized volume change}\left(6\text{ dpi}\right)=\frac{tumor\;volume\;\left(0\;dpi\right)-tumor\;volume\;\left(6\;dpi\right)}{tumor\;volume\;\left(0\;dpi\right)}, \end{array}$$

(5) $$\begin{array}{c} \text{Normalized volume change }\left(16,17\text{ dpi}\right)=\frac{tumor\;volume\;\left(0\;dpi\right)-tumor\;volume\;\left(16\text{ or }17\;dpi\right)}{tumor\;volume\;\left(0\;dpi\right)}, \end{array}$$

(6) $$\begin{array}{c} \text{Growth ratio }\left(x\;dpi\right)=\frac{tumor\;volume\;at\;x\;dpi}{tumor\;volume\;at\;0\;dpi}. \end{array}$$

The early SUVmax reduction% calculation was the earliest measurement after BCT; thus, SUVmax at 2 dpi was used for trial A and SUVmax at 1 dpi for trial B. The same acquisition times were used for early SUVmean reduction%. To analyze the effects of BCT, early (6 dpi) and late (16 or 17 dpi) time points were used, and from growth graphs, the early time point (6 dpi) was used. The late time point (16 or 17 dpi) was also used as it was the longest follow-up time with the shortest interval between A and B trials.

### 2.7. Experiment for Bacterial Load/Colonization Efficiency

For the evaluation of efficiency of bacterial load, an additional experiment was done after trials A and B. CT26 xenografts were generated in 9 mice. SL*Δ*ppGpp/lux was injected into tumor-bearing mice in the same way. *In vivo* BLi was done at 1 dpi in mouse 1~3, at 3 dpi in mouse 4~6, and at 5 dpi in mouse 7~9. After *in vivo* BLi, the blood, lung, liver, spleen, and tumor of each mice were extracted and imaged by ex vivo BLi.

### 2.8. Statistical Analysis

Spearman's rank correlation coefficient (rho) was used to analyze correlations between nonparametric variables. All statistical analyses were performed using Medcalc® v.18.5. Statistical significance was determined at *p* < 0.05.

## 3. Results

### 3.1. General Features before BCT

Between trials A and B, we observed no significant differences in tumor volume (0 dpi). Among the 11 mice, two died during the study: A5 at 2 dpi and A3 at 16 dpi (size measurements were performed until 12 dpi). Tumor volume at 0 dpi was not correlated with SUVmax (*p* > 0.5) or SUVmean (*p* > 0.5) at 0 dpi.

### 3.2. Tumor Growth and BLi after BCT

**Figure 2:**
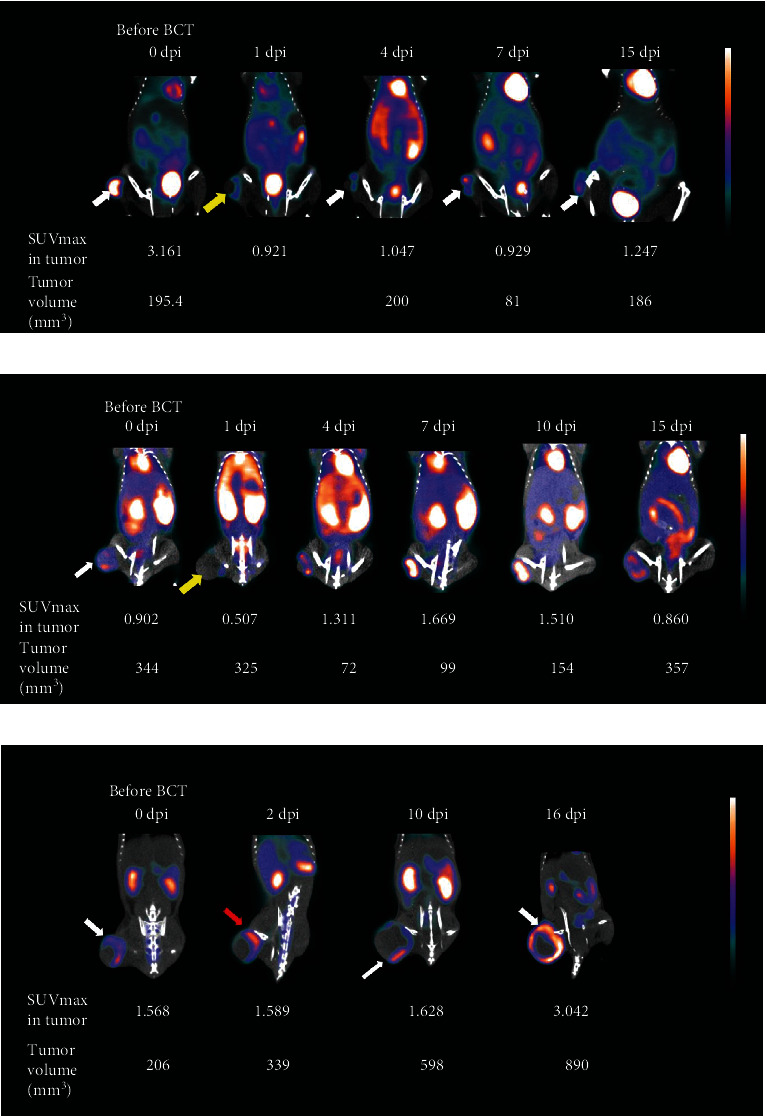
Three representative FDG PET/CT images after bacterial cancer therapy (BCT) using engineered *Salmonella typhimurium*. A tumor was generated in the right thigh of each mouse (BALB/c female mice, 6-weeks-old) using murine CT26 colon adenocarcinoma cells. After the tumor volume reached at least 180 mm^3^, BCT was performed by the intravenous injection of bacterial cells via the tail vein (bacterial dose = 4.5 × 10^7^ CFU/mouse in 100 *μ*L PBS). FDG PET/CT images were acquired before and after BCT. Coronal PET/CT fusion images are shown. FDG uptake was measured as SUVmax. White arrows indicate FDG uptake in tumors before BCT (a–c). Yellow arrows (a & b) indicate decreased FDG uptake 1 day after BCT. The red arrow (c) indicates a tumor showing no decrease in FDG uptake after BCT. Abbreviations: FDG, 2-deoxy-2[^18^F]fluoro-D-glucose; dpi, days post-injection; SUVmax, maximum standardized uptake value; SUVmean, mean standardized uptake value; PBS, phosphate-buffered saline. CFU, colony-forming units.

**Figure 3:**
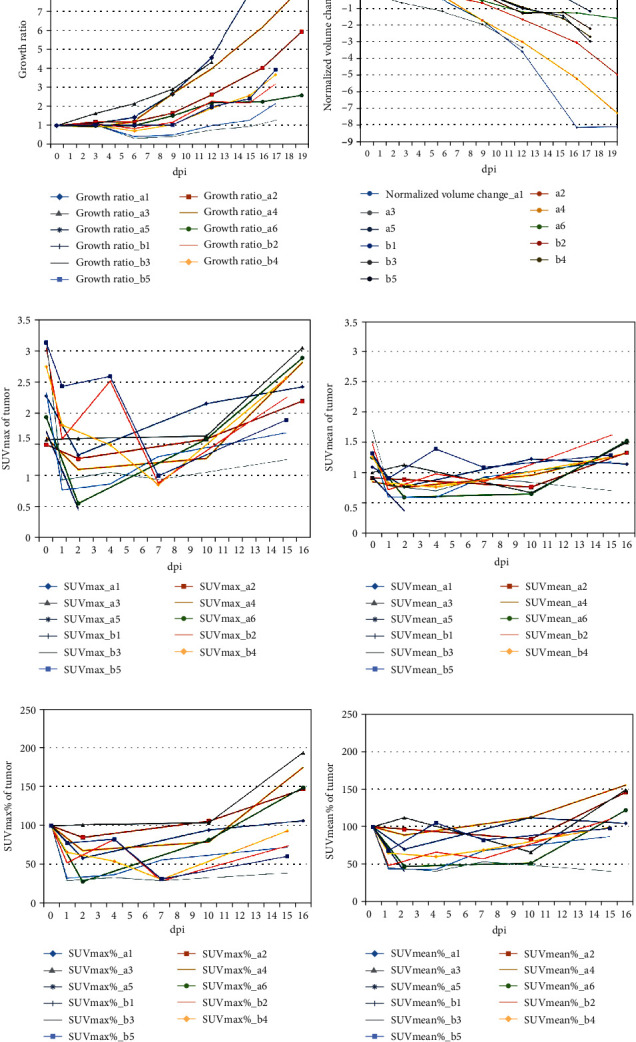
Changes in tumor volume and FDG uptake after bacterial cancer therapy (BCT) with engineered *Salmonella typhimurium* in a CT26 colon cancer cell line mouse model. Changes in tumor growth ratios (a), normalized tumor volume change (b), SUVmax (c), SUVmean (d), SUVmax% (e), and SUVmean% (f). Each point in the graphs represents a data measurement at that time point. (a) The tumor growth ratio was calculated as the measured volume divided by the initial volume (0 dpi). (b) Normalized volume change is calculated using tumor volume (0 dpi) − tumor volume (x dpi)/tumor volume (0 dpi) (c, d) Changes in FDG uptake in the tumor are shown as SUVmax in (c) and SUVmean in (d). (e, f) Percent changes in FDG uptake are shown as SUVmax% in (e) and SUVmean% in (f). SUVmax% and SUVmean% are calculated as 100 × SUV (specific dpi)/SUV (0 dpi). Abbreviations: FDG, 2-deoxy-2[^18^F]fluoro-D-glucose; dpi, days post-injection; SUVmax, maximum standardized uptake value; SUVmean, mean standardized uptake value.

*In vivo* BLi confirmed successful SL*Δ*ppGpp/lux accumulation in all mice tumors. After BCT, tumor growth was suppressed until 6–9 dpi in most mice, with growth then recommencing (Figures 2 and 3). However, in a1 and a3 mice, tumors were not suppressed and continued to grow (Figure 2(a)).

*In vivo* BLi detected SL*Δ*ppGpp/lux accumulation signal in tumors until 10 dpi, whereas no signals were detected at 15 or 16 dpi.In trial B, counting by optical signal was performed. Signals from *in vivo* BLi (1 dpi) correlated with FDG uptake in the tumor before BCT (SUVmax 0 dpi, rho = 0.95 and *p* = 0.0001; with SUVmean 0 dpi, rho = 0.67 and *p* = 0.0002). However, optical signals from *in vivo* BLi were not correlated with tumor volume (0 dpi), volume change, tumor growth ratio, early SUVmax reduction%, or early SUVmean reduction% (*p* > 0.1).

In a separate experiment, we enumerated bacterial accumulation in tumor after intravenous injection of SL*Δ*ppGpp/lux (4.5 × 10^7^ CFU/mouse). More than 10^8^ CFU/gram of SL*Δ*ppGpp/lux were accumulated in the tumor at 1, 3, and 5 dpi (Figure S1, electronic supplementary material (ESM)).

### 3.3. FDG Uptake Patterns in Tumors after BCT

FDG uptake in tumors decreased in the early days after BCT (1 or 2 dpi) in 10 of 11 mice, but it increased in later days. In only one mouse (a3), FDG uptake did not decrease in the early days after BCT (Figure 3(c)).

Between trials A and B, we observed no significant differences in hepatic FDG uptake and lung FDG uptake (Figure S2, ESM).

### 3.4. Correlations between FDG Uptake before BCT and Treatment Responses

**Figure 4:**
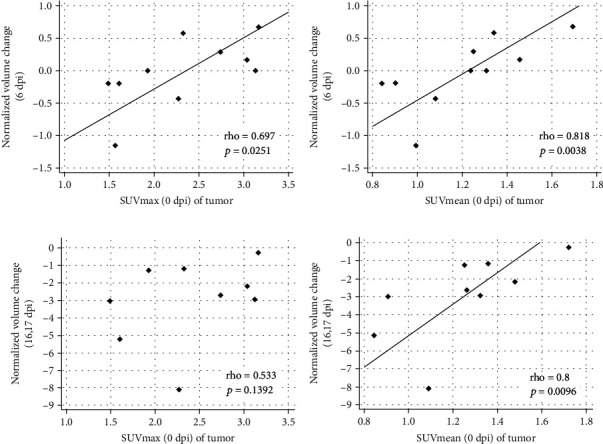
Correlations between treatment responses and pretreatment tumor FDG uptake in the CT26 colon cancer cell line mouse model. FDG uptake was measured as SUVmax and SUVmean. Data at 0 dpi were presented as mean data before BCT. Treatment responses were determined at two time points: early (6 dpi) and late (16 or 17 dpi). Normalized volume change is calculated using tumor volume (0 dpi) − tumor volume (×dpi)/tumor volume (0 dpi). (a) Tumor SUVmax at 0 dpi and normalized volume change (6 dpi). (b) Tumor SUVmean at 0 dpi and normalized volume change (6 dpi). (c) SUVmax at 0 dpi and normalized volume change at 16 or 17 dpi. (d) SUVmean at 0 dpi and normalized volume change at 16 or 17 dpi. Footnote: Of the 11 mice, two died during studies; A5 at 2 dpi and A3 at 16 dpi (size measurements were performed until 12 dpi). Therefore, the number of mice for (a) and (b) was 10 and for (c) and (d), 9. Abbreviations: FDG, 2-deoxy-2[^18^F]fluoro-D-glucose; dpi, days post-injection; SUVmax, maximum standardized uptake value; SUVmean, mean standardized uptake value.

During treatment responses, we analyzed normalized tumor volume change (Figure 4). At 6 dpi, both pre-BCT SUVmax and SUVmean indices in tumors correlated with normalized volume change after BCT (rho = 0.697 and *p* = 0.0251 and rho = 0.818 and *p* = 0.0038, respectively, Figures 4(a) and 4(b)). This meant that the higher the FDG uptake before BCT, the greater the tumor suppression. However, at 16 or 17 dpi, only the SUVmean value (rho = 0.8, *p* = 0.0096) was correlated with a normalized tumor volume change (Figures 4(c) and 4(d)).

Tumor volume at 0 dpi did not correlate with normalized tumor volume change at 6 dpi (*p* > 0.5) or at 16 or 17 dpi (*p* > 0.5, Figure S3, ESM). Neither SUVmax nor SUVmean at 0 dpi was correlated with tumor volume at 6 dpi (*p* > 0.4) or at 16 or 17 dpi (*p* > 0.05, Figure S4, ESM).

### 3.5. Correlations between FDG Uptake after BCT and Treatment Responses

**Figure 5:**
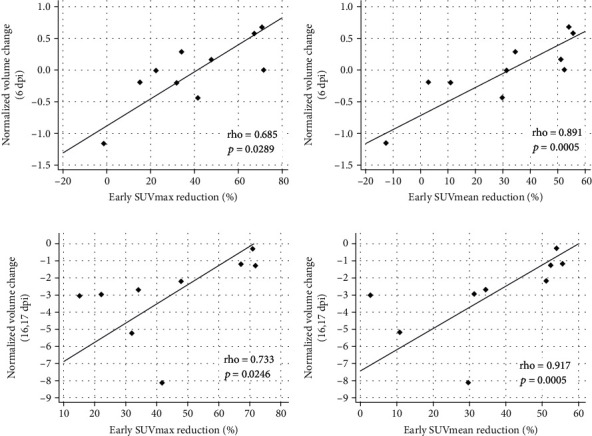
Correlations between treatment responses and early FDG uptake changes after bacterial cancer therapy (BCT) using engineered *Salmonella typhimurium* in a CT26 colon cancer cell line mouse model. Treatment responses were determined at two time points: early (6 dpi) and late (16 or 17 dpi). Normalized tumor volume change is calculated using tumor volume (0 dpi) − tumor volume (x dpi)/tumor volume (0 dpi). Early SUV reduction% was defined as 100 × (SUV at 0 dpi − SUV at 1 or 2 dpi)/SUV at 0 dpi. A higher early SUV reduction% meant a larger decrease in FDG uptake after BCT (1 or 2 dpi). (a) Early SUVmax reduction% and normalized volume change at 6 dpi. (b) Early SUVmean reduction% and normalized volume change at 6 dpi. (c) Early SUVmax reduction% and normalized volume change at 16 or 17 dpi. (d) Early SUVmean reduction% and normalized volume change at 16 or 17 dpi. Abbreviations: FDG, 2-deoxy-2[^18^F]fluoro-D-glucose; dpi, days post-injection; SUVmax, maximum standardized uptake value; SUVmean, mean standardized uptake value; early SUVmax reduction%, 100 × (SUV max at 0 dpi − SUV max at 1 or 2 dpi)/SUVmax at 0 dpi; early SUVmean reduction%, 100 × (SUV mean at 0 dpi − SUV max at 1 or 2 dpi)/SUVmean at 0 dpi.

After BCT, FDG uptake in tumors decreased in the early days (1 or 2 dpi). We calculated this using early SUVmax reduction% and early SUVmean reduction%. We also observed a significant correlation between normalized tumor volume change and early SUV reduction% (Figure 5). At the early response time point (6 dpi), both early SUVmax reduction% and early SUVmean reduction% were correlated with normalized tumor volume change (rho = 0.685 and *p* = 0.0289 for SUVmax reduction% and rho = 0.891 and *p* = 0.0005 for SUVmean reduction%; Figures 5(a) and 5(b)). At the late response time point (16 or 17 dpi), both early SUVmax reduction% and early SUVmean reduction% were correlated with normalized tumor volume change (rho = 0.733 and *p* = 0.0246 for SUVmax reduction% and rho = 0.917 and *p* = 0.0005 for SUVmean reduction%; Figures 5(c) and 5(d)). Taken together, these data indicated that a reduction in FDG uptake in the early days after BCT predicted BCT outcomes.

From tumor volume analysis at 6 dpi, both early SUVmax reduction% (rho = −0.738 and *p* = 0.0149) and early SUVmean reduction% (rho = −0.817 and *p* = 0.0039) were negatively correlated with tumor volume. From analyses at 16 or 17 dpi, both early SUVmax reduction% (rho = −0.733 and *p* = 0.0246) and early SUVmean reduction% (rho = −0.833 and *p* = 0.0053) were negatively correlated with tumor volume (Figure S5, ESM).

## 4. Discussion

To our knowledge, this is the first pilot study to analyze FDG uptake changes in tumors after BCT. We observed several unique FDG uptake features: (1) Tumor FDG uptake decreased in the first 2 days after BCT and then increased, before tumor graft re-growth; (2) FDG uptake before BCT was correlated with normalized tumor volume change after BCT; and (3) early FDG reduction% after BCT was correlated with normalized tumor volume change after BCT.

The mechanisms underlying tumor-targeting bacteria may involve the chemotactic system [32–34], inflammatory cytokine-mediated dilation of tumor blood vessels [35, 36], and immune-privileged tumor environments [7, 37, 38]. However, the mechanism underpinning the therapeutic effects of BCT using *Salmonella* species is unclear. *Salmonella* species are believed to kill tumor cells by apoptosis and/or autophagy via alterations in host antitumor immune responses or nutrient deprivation [1]. *Salmonella* also activates the inflammasome pathway via damage signal release from cancer cells and macrophages [39]. These effects may contribute to the BCT therapeutic effects of *Salmonella*.

FDG is not a specific radiotracer for apoptosis. However, BCT mechanisms are not simply limited to apoptosis. A recent study investigating apoptosis imaging tracers reported that FDG was more reliable and sensitive for evaluating therapeutic effects [40]. The authors of this study reported that tumor apoptosis, induced by an antiangiogenic agent, was more sensitively and reliably monitored by FDG when compared with Annexin V-based apoptosis imaging [40]. Furthermore, FDG uptake in malignant tumors was influenced not only by cancer cells but also cells in the tumor microenvironment [41–43]. Activated neutrophils increased GLUT type 3 and 4 expression resulting in increased glucose uptake [42], and tumor necrosis factor-*α* secreted by macrophages increased FDG uptake in tumor cells [43]. Lymphocytes also increased FDG uptake in tumors based on their numbers and activation status [41]. Therefore, elevated FDG accumulation in tumors was reflected by the high numbers and activities of both cancer and immune cells. Based on these proposed uptake mechanisms, FDG is a good candidate for evaluating BCT.

We identified an early (within 1 or 2 days) decrease in FDG uptake in tumors after BCT. This reflected *Salmonella* actions in the early period; bacterial accumulation in tumors was confirmed by *in vivo* BLi at 1 dpi. Ganai et al. reported that *S. typhimurium* accumulated in tumors 3 hours after systemic injection [32], and at up to 48 hours later, tumor apoptosis had increased and viable tissue decreased [32]. These data were consistent with our findings of an early decrease in FDG uptake in tumors after BCT.

Previous studies also reported that high tumor FDG uptake was associated with poor survival in patients with lung cancer, breast cancer, colon cancer, or lymphoma [44]. In addition, tumor FDG uptake correlated with the levels of several prognostic factors such as p53, Ki67, GLUT1, and hexokinase in patients with colon cancer [45]. In contrast, in this study, tumors with high FDG uptake before BCT showed better results after BCT. We observed that FDG uptake before BCT correlated with normalized tumor volume changes after BCT. High FDG uptake before BCT showed better results after BCT and suggested BCT was more effective in tumors with increased glucose metabolism. This discrepancy was understandable due to the important role of immune cells in the tumor microenvironment in BCT when compared with conventional treatments, such as chemotherapy and radiotherapy. In BCT, activated immune cells are mainly involved in tumoricidal mechanisms [1, 39]. A high FDG uptake represents high numbers of activated immune cells [46, 47]. In previous studies, we also reported that BCT outcomes correlated well with immune cell infiltration and activation in the tumor milieu [8, 23]. Our findings also indicated different treatment mechanisms between conventional cancer therapies and BCT. Additionally, when we analyzed *in vivo* BLi optical signals in trial B, they were positively correlated with FDG uptake in the tumor before BCT, suggesting that tumor targeting by *S. typhimurium* was better in hypermetabolic tumors.

We observed that early SUV reduction% correlated with normalized tumor volume change after BCT. This meant that a higher reduction in FDG uptake in the early days after BCT predicted a smaller tumor mass after BCT. We hypothesized that early bacterial reactions could determine the final effects of BCT and this information could be used to predict BCT effects.

This pilot study had some limitations. The number of study animals was small. Further imaging studies are underway to compare the characteristics of different radiotracers such as FDG and fluorodeoxysorbitol in BCT. Also, we did not perform *in vitro* histological and genomic analyses before and after BCT. However, our study was exclusively focused on the analysis of FDG uptake patterns in BCT. Further research investigating *in vitro* molecular genomic changes following BCT is warranted.

## 5. Conclusions

This was the first study investigating FDG uptake patterns in BCT using attenuated *S. typhimurium*. Early after BCT, FDG tumor uptake decreased and then increased at later stages. The higher the FDG uptake before BCT, the better the BCT response, and FDG uptake patterns were related to tumor volume change after BCT. Therefore, FDG uptake appears to be a good candidate for BCT evaluating BCT.

## Data Availability

The data is available on request.

## Supplementary Materials

Supplementary Materials Figure S1: accumulation of engineered *Salmonella typhimurium* in a CT26 colon cancer model. Tumor targeting of engineered *S. typhimurium* (SLppGpp-lux) in a CT26 colon cancer model was confirmed by *in vivo* BLi from 1 dpi after BCT. Data are showing bacterial load/colonization efficiency at the tumor after BCT in a CT26 colon cancer model. Figure S2: comparison of tumor volume and liver and lung FDG uptake before BCT in a CT26 colon cancer model between trial A and B. Figure S3: correlation between pre-treatment tumor volume and tumor FDG uptake before treatment and treatment results in a CT26 colon cancer model. Figure S4: correlation between pretreatment FDG uptake and tumor volume after BCT in a CT26 colon cancer model. Figure S5: correlation between early SUV reduction% and tumor volume after BCT in a CT26 colon cancer model. Figure S6: changes in FDG uptake in liver and lung before and after BCT in a CT26 colon cancer model.
