# Supplementary material for: Pattern of F-18 FDG Uptake in Colon Cancer after Bacterial Cancer Therapy Using Engineered Salmonella Typhimurium: A Preliminary In Vivo Study
Source: Mol Imaging. 2022 Apr 19;2022:9222331. doi: 10.1155/2022/9222331 (PMC9042370; doi:10.1155/2022/9222331)
Supplement: Supplementary Materials — Figure S1: accumulation of engineered Salmonella typhimurium in a CT26 colon cancer model. Tumor targeting of engineered S. typhimurium (SLppGpp-lux) in a CT26 colon cancer model was confirmed by in vivo BLi from 1 dpi after BCT. Data are showing bacterial load/colonization efficiency at the tumor after BCT in a CT26 colon cancer model. Figure S2: comparison of tumor volume and liver and lung FDG uptake before BCT in a CT26 colon cancer model between trial A and B. Figure S3: correlation between pre-treatment tumor volume and tumor FDG uptake before treatment and treatment results in a CT26 colon cancer model. Figure S4: correlation between pretreatment FDG uptake and tumor volume after BCT in a CT26 colon cancer model. Figure S5: correlation between early SUV reduction% and tumor volume after BCT in a CT26 colon cancer model. Figure S6: changes in FDG uptake in liver and lung before and after BCT in a CT26 colon cancer model. [file 9222331.f1.docx]

***Electronic Supplementary material***

**Title: Pattern of F-18 FDG uptake in colon cancer after bacterial cancer therapy using engineered Salmonella typhimurium: a preliminary in vivo study**

**Short title: Pattern of F-18 FDG uptake after bacterial cancer therapy**

Ari Chong^1, 2^, Dinh-Huy Nguyen^2,3^, Hyeon Sik Kim^4^, June-Key Chung^2,5^, Jung-Joon Min^2,3^

^1^Department of Nuclear Medicine, Chosun University Medical School, Gwangju, South Korea.

^2^Department of Nuclear Medicine, Chonnam National University Medical School, Jeonnam 519-763, South Korea.

^3^Department of Nuclear Medicine, Chonnam National University Hwasun Hospital, Jeonnam 519-763, South Korea.

^4^Medical & Bio Photonics Research Center, Korea Photonics Technology Institute, Gwangju, South Korea.

^5^Department of Nuclear Medicine, Seoul National University College of Medicine.

**Corresponding author**

**Jung-Joon Min**

jjmin@jnu. ac.kr

Professor and Chair, Department of Nuclear Medicine, Chonnam National University Medical School & Hwasun Hospital, Jeonnam 519-763, South Korea.


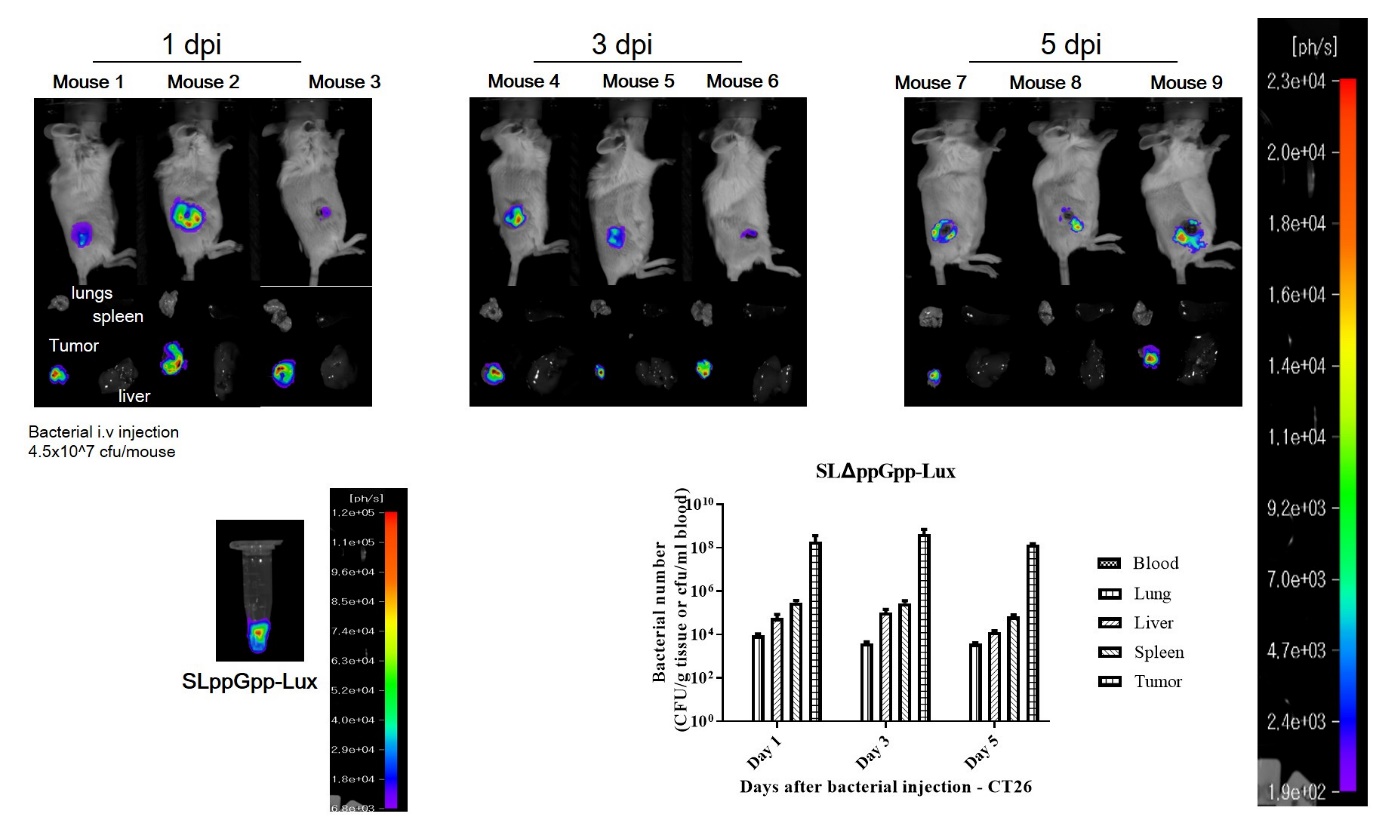


**Figure S1.** **Accumulation of engineered *Salmonella typhimurium* in a CT26 colon cancer model.**

Tumor targeting of engineered *S. typhimurium (*SLppGpp-lux) in a CT26 colon cancer model was confirmed by *in vivo* BLi from 1 dpi after BCT. Data are showing bacterial load/colonization efficiency at the tumor after BCT in a CT26 colon cancer model.

Abbreviations: *in vivo* BLi, *in vivo* bioluminescence imaging; BCT, bacterial cancer therapy; SLppGpp-lux, Attenuated S. typhimurium defective in ppGpp synthesis (RelA::cat, SpoT::kan) and expressing the bacterial luciferase (lux) operon (SHJ2037); CFU, colony-forming units; CT26, murine CT26 colon adenocarcinoma cell line ; dpi, days post-injection


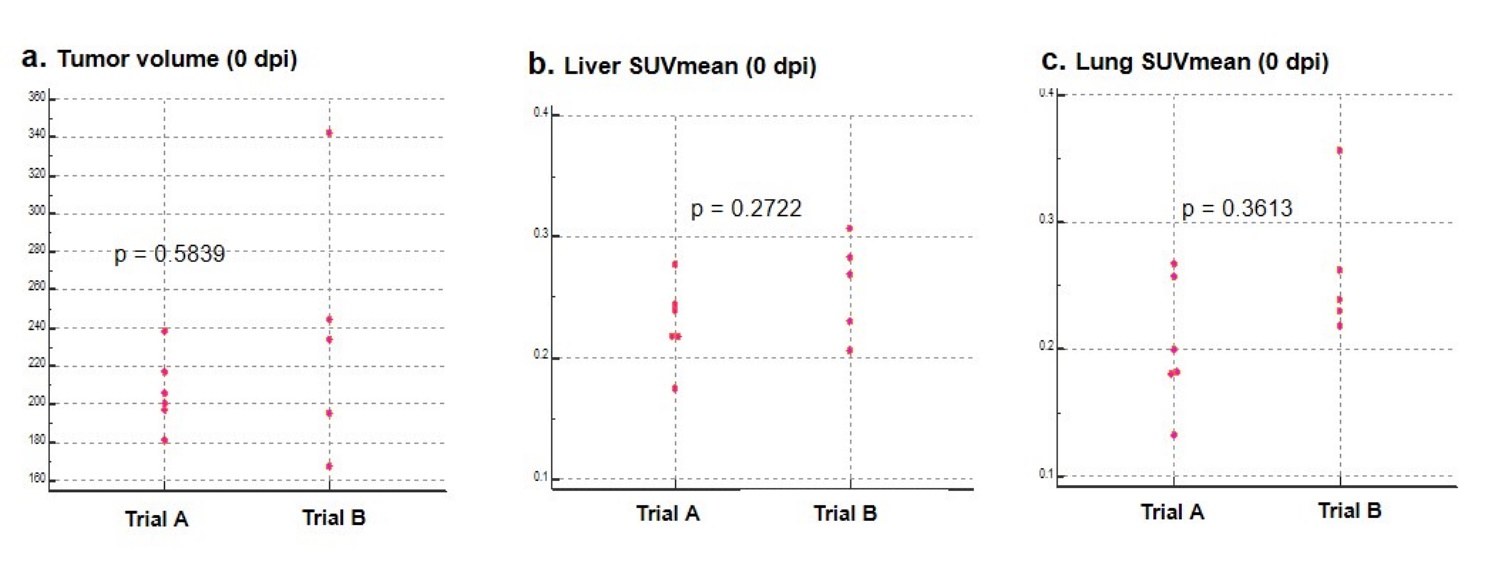


**Figure S2. Comparison of tumor volume and liver and lung FDG uptake before BCT in a CT26 colon cancer model between trial A and B**

Abbreviations: BCT, bacterial cancer therapy; dpi, days post-injection; SUVmax, maximum standardized uptake value; SUVmean, mean standardized uptake value.

**
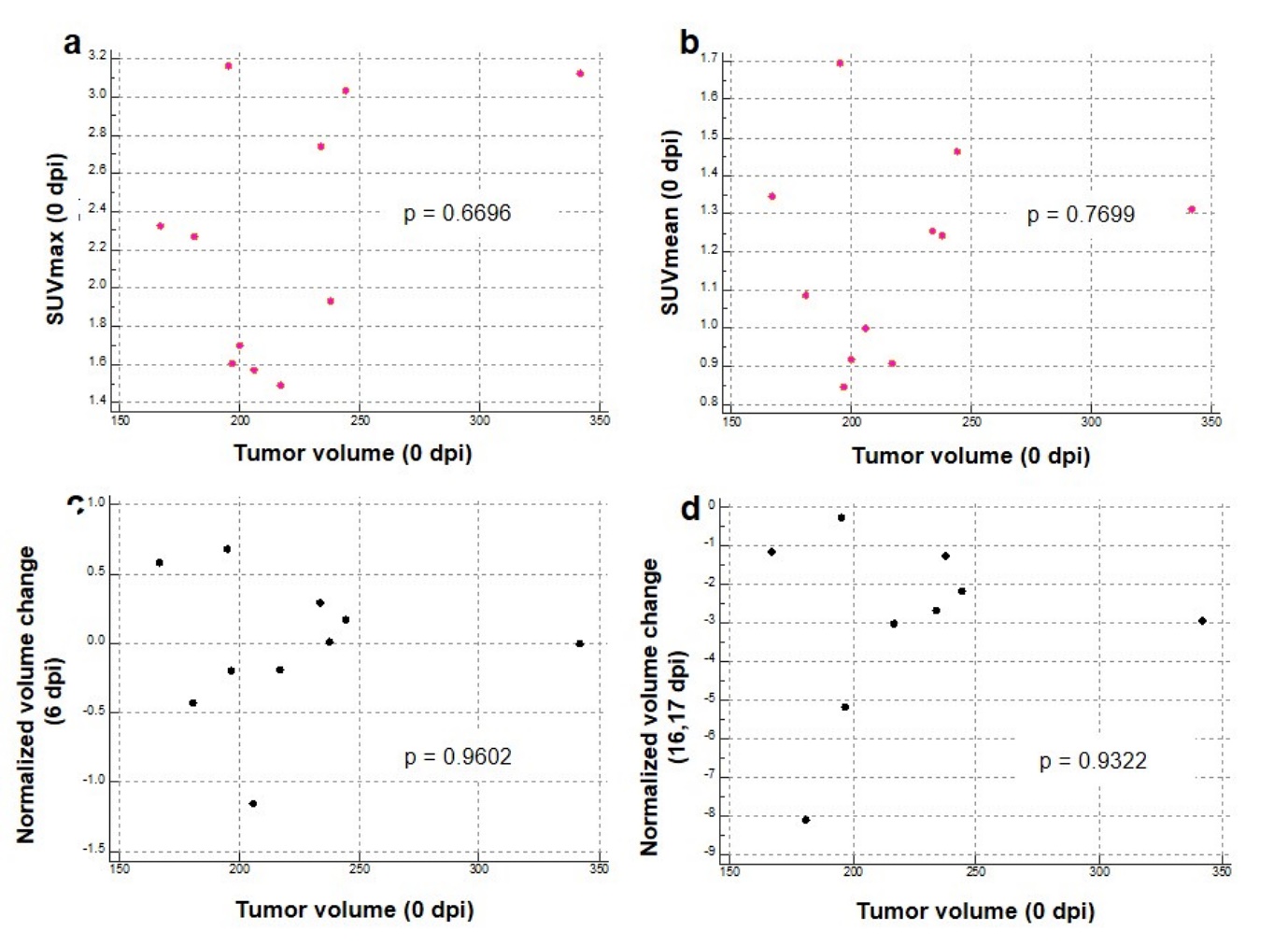
**

**Figure S3. Correlation between pre-treatment tumor volume and tumor FDG uptake before treatment and treatment results in a CT26 colon cancer model.**

Tumor volume (0 dpi) was measured before BCT with engineered *Salmonella typhimurium* in a CT26 colon cancer model.

Rank correlation analysis was performed between tumor volume at 0 dpi and (a) tumor SUVmax at 0 dpi, (b) tumor SUVmean at 0 dpi, (c) normalized tumor volume change at 6 dpi, and (c) normalized tumor volume change at 16 or 17 dpi.

Abbreviations: FDG, 2-deoxy-2[^18^F]fluoro-D-glucose; dpi, days post-injection; SUVmax, maximum standardized uptake value; SUVmean, mean standardized uptake value.

**
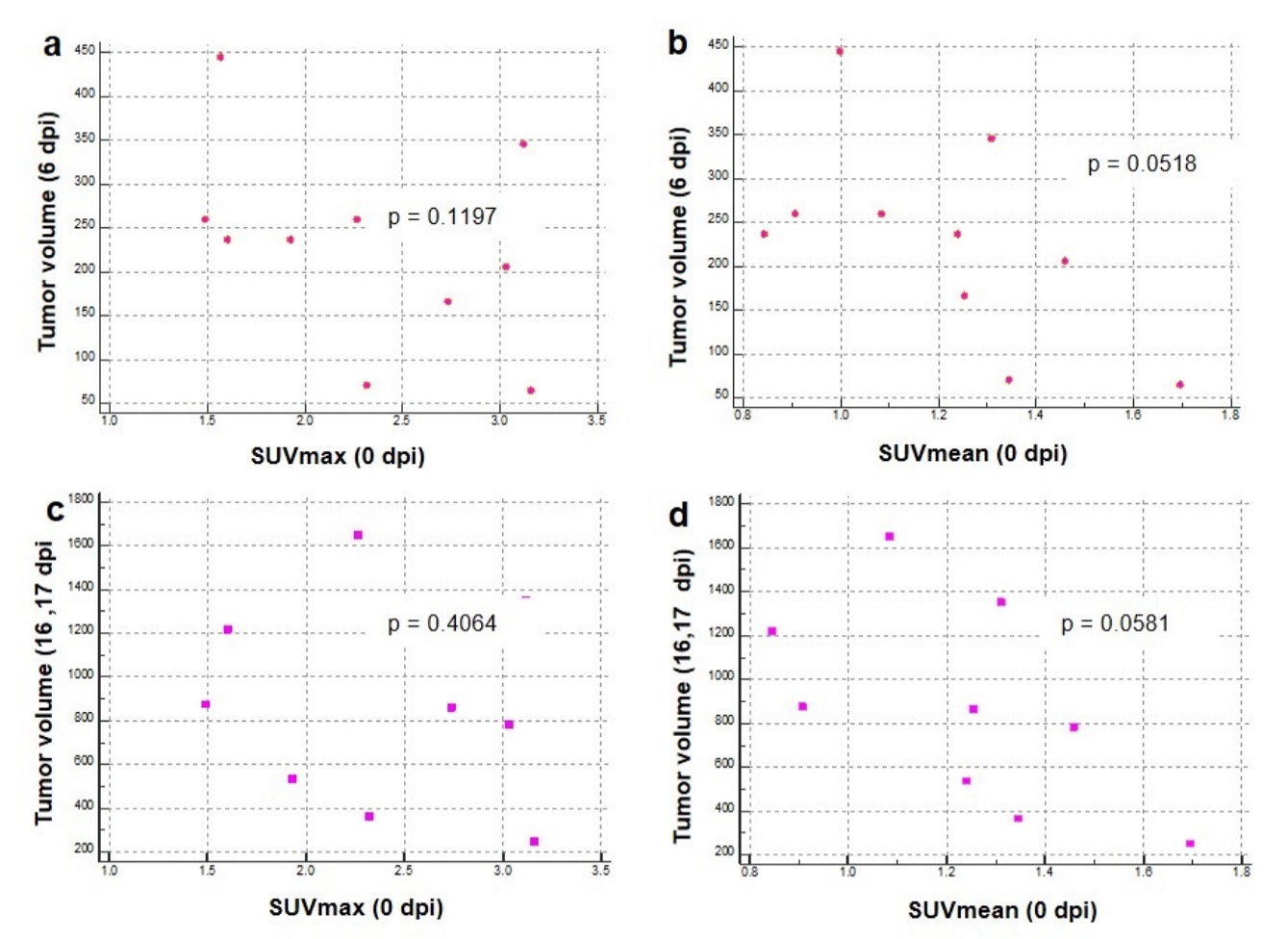
**

**Figure S4. Correlation between pre-treatment FDG uptake and tumor volume after BCT in a CT26 colon cancer model.**

Rank correlation analysis was performed between (a) SUVmax at 0 dpi and tumor volume at 6 dpi, (b) SUVmean at 0 dpi and tumor volume at 6 dpi, (c) SUVmax at 0 dpi and tumor volume at 16 or 17 dpi, and (d) SUVmean at 0 dpi and tumor volume at 16 or 17 dpi.

Abbreviations: FDG, 2-deoxy-2[^18^F]fluoro-D-glucose; dpi, days post-injection; SUVmax, maximum standardized uptake value; SUVmean, mean standardized uptake value.


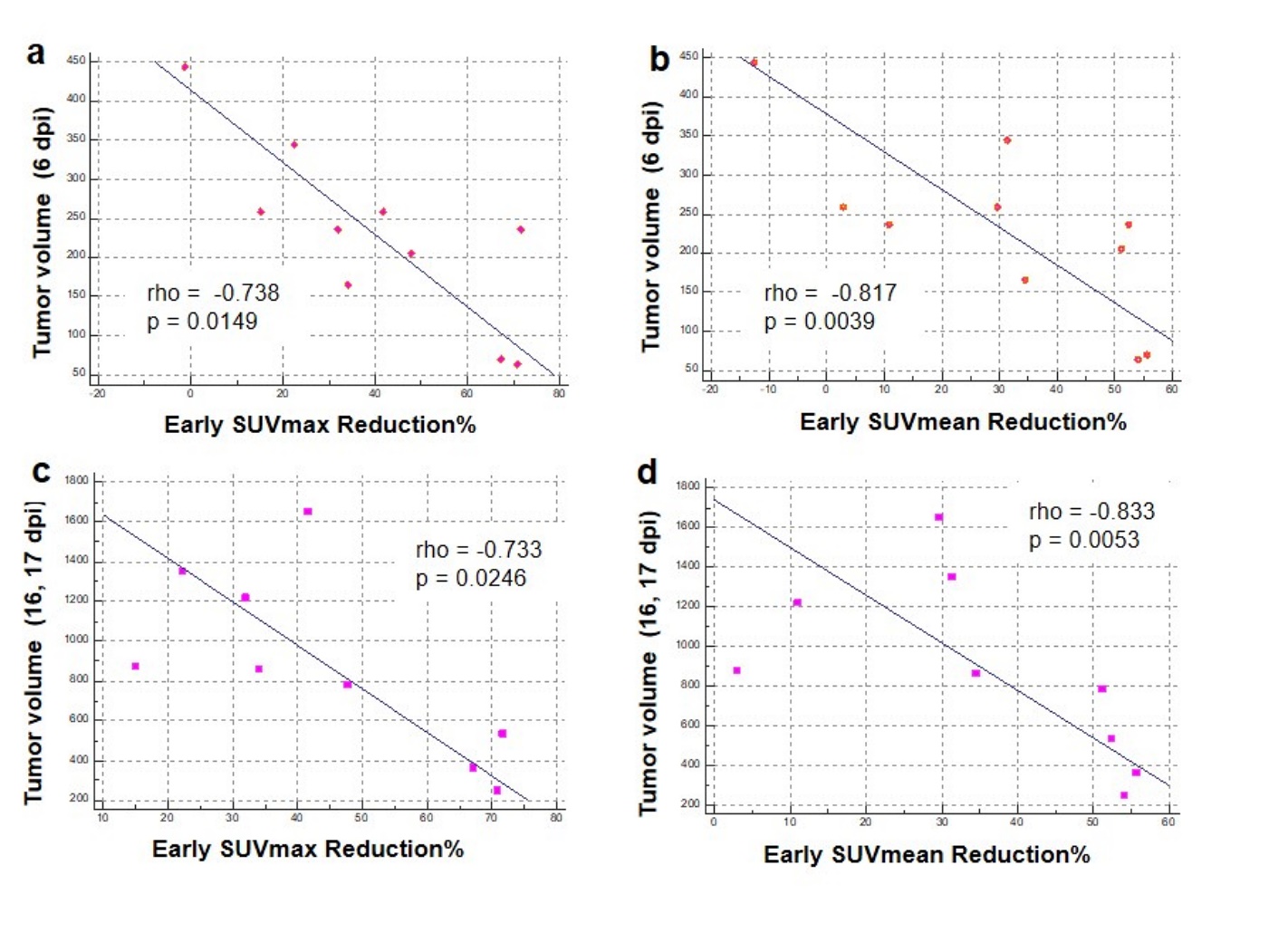


**Figure S5. Correlation between early SUV reduction% and tumor volume after BCT in a CT26 colon cancer model.**

Rank correlation analysis was performed between (a) early SUVmax reduction% and tumor volume at 6 dpi, (b) early SUVmean reduction% and tumor volume at 6 dpi, (c) early SUVmax reduction% and tumor volume at 16 or 17 dpi, and (d) early SUVmean reduction% and tumor volume at 16 or 17 dpi. Early SUV reduction% was defined as 100 × (SUV (0 dpi) - SUV (1 or 2 dpi)/SUV (0 dpi).

Abbreviations: dpi, days post-injection; SUVmax, maximum standardized uptake value; SUVmean, mean standardized uptake value.


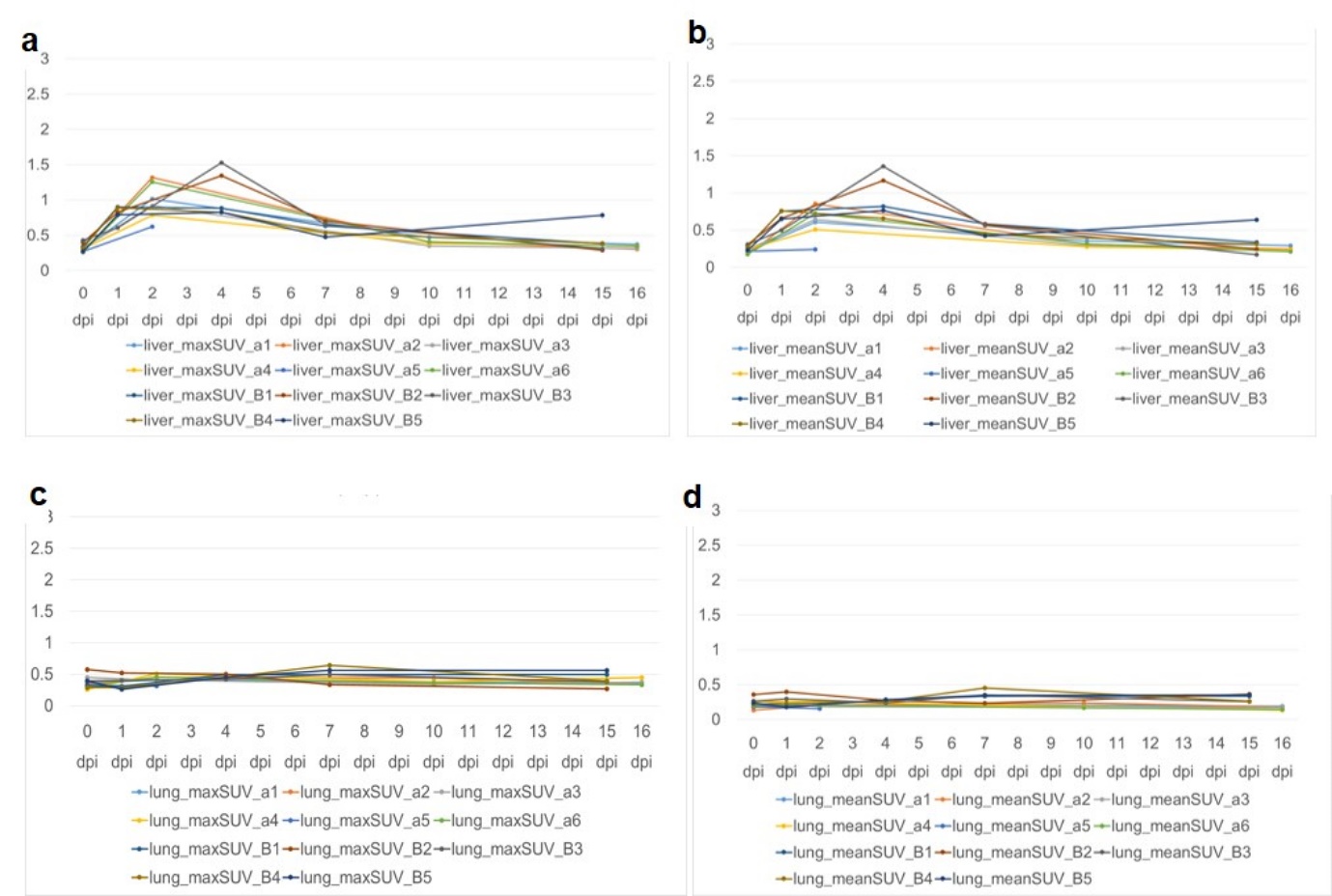


**Figure S6.** Changes in FDG uptake in liver and lung before and after BCT in a CT26 colon cancer model.

Abbreviations: BCT, bacterial cancer therapy; dpi, days post-injection; SUVmax, maximum standardized uptake value; SUVmean, mean standardized uptake value.
